# Genetic Dissection of Germinability under Low Temperature by Building a Resequencing Linkage Map in *japonica* Rice

**DOI:** 10.3390/ijms21041284

**Published:** 2020-02-14

**Authors:** Shukun Jiang, Chao Yang, Quan Xu, Lizhi Wang, Xianli Yang, Xianwei Song, Jiayu Wang, Xijuan Zhang, Bo Li, Hongyu Li, Zhugang Li, Wenhua Li

**Affiliations:** 1 Crop Cultivation and Tillage Institute of Heilongjiang Academy of Agricultural Sciences, Heilongjiang Provincial Key Laboratory of Crop Physiology and Ecology in Cold Region, Heilongjiang Provincial Engineering Technology Research Center of Crop Cold Damage, Harbin 150086, China; wanglizhi0451@163.com (L.W.); aiwei.ni@163.com (X.Y.); xijuanzhang@163.com (X.Z.); blnky@163.com (B.L.); 2 Institute of Genetics and Developmental Biology, Chinese Academy of Sciences, Beijing 100101, China; chaoyang@genetics.ac.cn (C.Y.); xwsong@genetics.ac.cn (X.S.); 3 Rice Research Institute of Shenyang Agricultural University, Shenyang 110866, China; kobexu34@syau.edu.cn (Q.X.); ricewjy@126.com (J.W.); 4 College of Agronomy, Heilongjiang Bayi Agricultural University, Daqing 163000, China; ndrice@163.com

**Keywords:** *japonica* rice, cold stress, germinability, high-density linkage map, QTLs

## Abstract

Among all cereals, rice is highly sensitive to cold stress, especially at the germination stage, which adversely impacts its germination ability, seed vigor, crop stand establishment, and, ultimately, grain yield. The dissection of novel quantitative trait loci (QTLs) or genes conferring a low-temperature germination (LTG) ability can significantly accelerate cold-tolerant rice breeding to ensure the wide application of rice cultivation through the direct seeding method. In this study, we identified 11 QTLs for LTG using 144 recombinant inbred lines (RILs) derived from a cross between a cold-tolerant variety, Lijiangxintuanheigu (LTH), and a cold-sensitive variety, Shennong265 (SN265). By resequencing two parents and RIL lines, a high-density bin map, including 2,828 bin markers, was constructed using 123,859 single-nucleotide polymorphisms (SNPs) between two parents. The total genetic distance corresponding to all 12 chromosome linkage maps was 2,840.12 cm. Adjacent markers were marked by an average genetic distance of 1.01 cm, corresponding to a 128.80 kb physical distance. Eight and three QTL alleles had positive effects inherited from LTH and SN265, respectively. Moreover, a pleiotropic QTL was identified for a higher number of erected panicles and a higher grain number on Chr-9 near the previously cloned *DEP1* gene. Among the LTG QTLs, *qLTG3* and *qLTG7b* were also located at relatively small genetic intervals that define two known LTG genes, *qLTG3-1* and *OsSAP16*. Sequencing comparisons between the two parents demonstrated that LTH possesses *qLTG3-1* and *OsSAP16* genes, and SN-265 owns the *DEP1* gene. These comparison results strengthen the accuracy and mapping resolution power of the bin map and population. Later, fine mapping was done for *qLTG6* at 45.80 kb through four key homozygous recombinant lines derived from a population with 1569 segregating plants. Finally, *LOC_Os06g01320* was identified as the most possible candidate gene for *qLTG6*, which contains a missense mutation and a 32-bp deletion/insertion at the promoter between the two parents. LTH was observed to have lower expression levels in comparison with SN265 and was commonly detected at low temperatures. In conclusion, these results strengthen our understanding of the impacts of cold temperature stress on seed vigor and germination abilities and help improve the mechanisms of rice breeding programs to breed cold-tolerant varieties.

## 1. Introduction

Rice (*Oryza sativa* L.), which is a staple food and nutritional source for many countries, fulfills the nutritional requirements for over half of the world’s population and is cultivated across the globe, except in a few areas where icy conditions prevail during most of the year [1]. Since rice originated in tropical and sub-tropical climates, it is one of the most sensitive cereals to cold stress [2], which limits its growth, development, and yield formation, especially when cold stress prevails at the germination stage [3]. Cold stress impacts all growth stages of rice, including tillering, booting, flowering, and grain-filling, but if stress dominates at the germination stage, it proves adverse for rice development at later growth stages. Low-temperature stress during the germination stages of rice affects seedling vigor and produces poor seedling emergence and an uneven stand establishment with a lower growth rate, which delays panicle development and enhances spikelet sterility [4]. Across China’s mainland, most of the rice cultivation areas are affected by frequent cold stresses. The Chinese agricultural sector suffers from an average loss of rice of about 3–5 million tons of rice every year due to these frequent cold stresses [5]. Two kinds of cold stresses occur in Chinese rice-growing regions. The (1) “cold spring” and (2) “cold autumn wind” often cause severe yield losses in double-cropping rice regions across the Yangtze River in China. In Northeast China (NEC), commonly considered a rice region at a high latitude, and the Yunnan-Guizhou Plateau, considered a rice cultivation region at a low latitude, severe cold summer damage was observed, with an average of three to four years of cold stress. These areas are expected to encounter more severe damage in the near future due to low temperature stress [6].

Traditional genetic and molecular analyses on Arabidopsis, rice, and other model plants have revealed that C-repeat binding factors (CBFs) are mainly involved in the cold signaling pathways. Recent studies have further revealed that the protein kinases and transcription factors are also involved in cold signaling in plants [7]. Additionally, genetic research on rice has detected numerous quantitative trait loci that control cold tolerance on nearly all 12 chromosomes [8]. Among these loci, only a few quantitative trait loci (QTLs) have been thoroughly researched and cloned, while the functional mechanisms of most are still largely unknown. Among all the QTLs for low-temperature germination in rice, only *qLTG3-1* and *OsSAP16* were cloned. *qLTG3-1* encodes a protein with glycine-rich and lipid trans-protein domain structures [9], and *OsSAP16* encodes a zinc-finger protein that positively regulates germination under low temperatures [10]. The QTLs *qCTS7*, *LTG1*, *COLD1*, *qCTS9*, *bZIP73*, *qPSR10*, and *HAN1* control the pathways for cold tolerance in rice. *qCTS7* increases cold tolerance at the seedling stage due to its overexpression [11]. *LTG1* encodes a casein kinase that plays a role in regulating the rice growth rate under cold stress [4]. A regulator of Ca^2+^ signaling in the plasma membrane and endoplasmic reticulum is encoded by *COLD1* [12], whereas a novel protein that interacts with Brassinosteroid Insensitive-1 is encoded by *qCTS9* [13]. There is a functional interaction between *bZIP73* and *bZIP71* that makes rice seedlings tolerant to greater cold [14]. A single-nucleotide polymorphism (SNP), SNP^2G^, at position 343 in *qPSR10*, is responsible for conferring cold tolerance during the seedling stage [15]. *HAN1* encodes an oxidase that provides functional contributions to the Jasmonic acid mediated cold response in temperate *japonica* rice [16]. The other three QTLs, *Ctb1*, *CTB4a*, and *bZIP73*, control cold tolerance at the booting stage. The first encodes an F-box protein [17], the second encodes a leucine rich repeat kinase that enhances seed setting through increased ATP-synthase activity under low temperature stress [3], and the third increases the cold tolerance rate by enhancing the soluble sugar transport from anthers to pollens at the booting stage [18].

Advances in genome-wide sequencing technology have provided an effective method to detect DNA sequencing differences among closely related rice materials and to ensure the presence of sufficient markers for a genetic mapping analysis. A genotype calling method for RILs that utilizes resequencing data was developed [19], which determined the construction of resequencing bin maps and accelerated genetics-based studies for many crops, including cereals [19,20,21,22,23,24,25,26,27]. Based on the above discussions, many important advances have been achieved in the study of rice cold stress, but we still need to use high-throughput sequencing technology to mine further cold-tolerance genes from *japonica* rice, especially cold tolerance genes at the germination stage for breeding practice.

The current study was arranged with the following objectives: (1) constructing a high density bin map by re-sequencing a set of 144 RILs with large differences in germination abilities under cold stress; (2) identifying QTLs for LTG in RIL populations by using the built linkage map; and (3) creating an accurate map of *qLTG6*, with a high low-temperature germinability (LOD) score and relatively small genetic intervals.

## 2. Results

### 2.1. Phenotypic Variation among the Parent and RIL Populations

**Figure 1:**
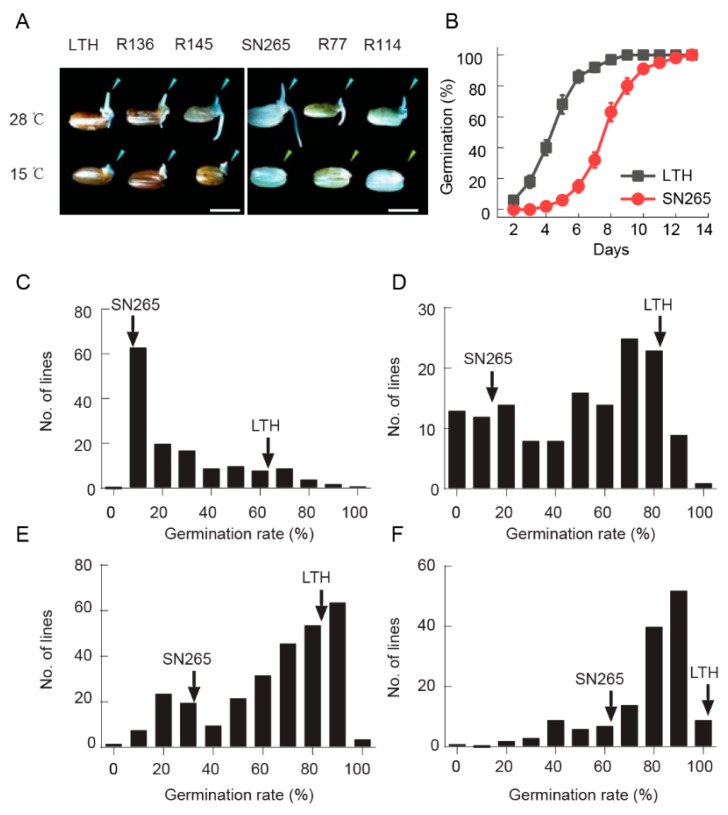
The seed germination of two parents and their recombinant inbred lines (RILs) at normal (28 °C) and low (15 °C) temperatures. (**A**). The germination phenotype of the two parents, Lijiangxintuanheigu (LTH) and Shennong265 (SN265), as well as the four RIL lines incubated for 3 days at 28 °C and for 6 days at 15 °C. R136 and R145 have 11 positive QTLs, whereas R77 and R144 have no positive QTLs. The bars = 5 mm; (**B**). The germination behavior of LTH (black) and SN265 (red) under low temperatures at 15 °C. The means are shown in triplicate; (**C**–**F**). The frequency distribution of low-temperature germinability in the RILs incubated for 5 days (**C**), 6 days (**D**), 7 days (**E**), and 8 days (**F**). Arrowheads indicate LTH or SN265.

After incubating at 28 °C for three days, the germination rates of LTH and SN265 were 100%, indicating that both parents share a similar germination rate at a normal temperature, as presented in Figure 1A. However, when they were incubated at 15 °C for six days to determine their low temperature germinability, LTH and SN265 showed broad differences in their germination rates, as shown in Figure 1A,B. There was a delay of about two days in germination after incubating LTH under low temperature conditions, and, thereafter, the germination rate reached up to 90% after five days of incubation. Comparatively, for SN265, germination started after five days of incubation and, thereafter, took five more days to reach 90% of the germination of LTH, which shows a clear difference in the time taken for germination by LTH and SN265 (Figure 1B). A range of five to eight days was chosen for a proper and dynamic comparison of the low-temperature germinability among the parents and RILs (Figure 1C–F). At all four time points, the distributions of the germination percentages of the RIL populations were continuous, indicating that low-temperature germinability is controlled by QTLs. Generally, it is already known that the longer the germination time, the higher the germination percentage. Therefore, on the basis of the larger differences in germination between the parents after six days (LTH and SN265 showed 86.5% and 15.4%, respectively), those data were used for the subsequent QTL mapping.

### 2.2. Bin Map Construction and Comparison of the Physical Map to the Genetic Map

**Figure 2:**
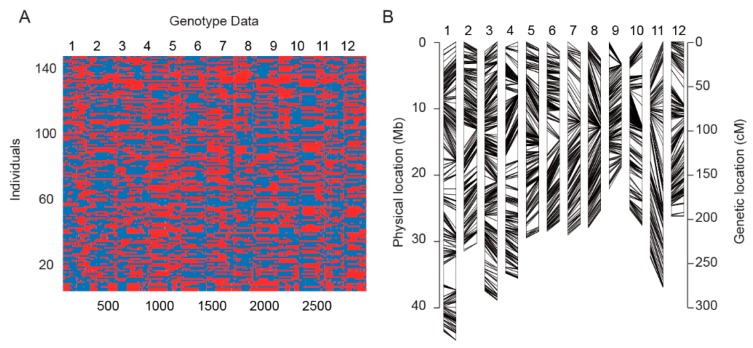
High-resolution genotyping and comparison of the physical maps and genetic maps of RILs. (**A**). Aligned recombination maps of 144 RILs from a cross between LTH and SN265. The two genotypes are indicated by blue (SN265) and red (LTH); (**B**). Comparison of the physical maps and genetic maps. Left: physical map. Right: genetic map.

For proper identification of the SNP between the two parents as molecular markers, deep resequencing was done for LTH and SN265. The effective sequencing depths of LTH and SN265 were about 19-fold and 17-fold, respectively. After resequencing, a total of 123,859 SNPs were produced between the two parents. Construction of the genetic linkage map for the RILs was carried out by re-sequencing the 144 RIL lines, which were already derived from LTH and SN265. In this way, these 144 RIL lines produced a range of reads between about 7,448,879 and 12,118,209, with a mean value of 9,660,250. The overall effective depth coverage of these RIL lines ranged from 5.98-fold to 9.73-fold, with an average depth of 7.75-fold. About 58,738 recombination breakpoints were used to construct the fine bin map. Each RIL line was comprised of breakpoints ranging from 236 to 1007 breakpoints, with an average value of 405. The 144 RIL lines were merged into a high-density bin map comprising 2,818 recombination bins of the 12 rice chromosomes, including most recombination events (Figure 2A). The average of the physical intervals for the adjacent bins was observed between 15.00 kb and 3.60 Mb, with a mean value of 128.80 kb, where most of the bins with physical intervals less than 100.00 kb were found to be around 68.60%. Overall, only 32 bins exceeded a physical interval of 1.00 Mb, most of which were in centromeric regions. The average physical distance between the bin markers was 98.23 kb and 169.03 kb on chromosome 10 and chromosome 1, respectively. Then, we constructed 12 chromosomes with a total genetic distance of 2,840.12 cm (Figure 2B). The average genetic distance observed between the two markers was nearly 1.01 cm. Among all chromosomes under consideration, chromosome 1 was the longest, with 254 bin markers, and its genetic distance was 338.65 cm. In contrast, chromosome 9 was seen to be the shortest one, with 188 bin markers encompassing a genetic distance of 111.32 cm, as given in Table 1. Comparing the genetic distance between chromosome 9 and 11, the average values were 0.59 cm and 1.74 cm, respectively.

### 2.3. The Quality and Accuracy of the Bin Map

**Figure 3:**
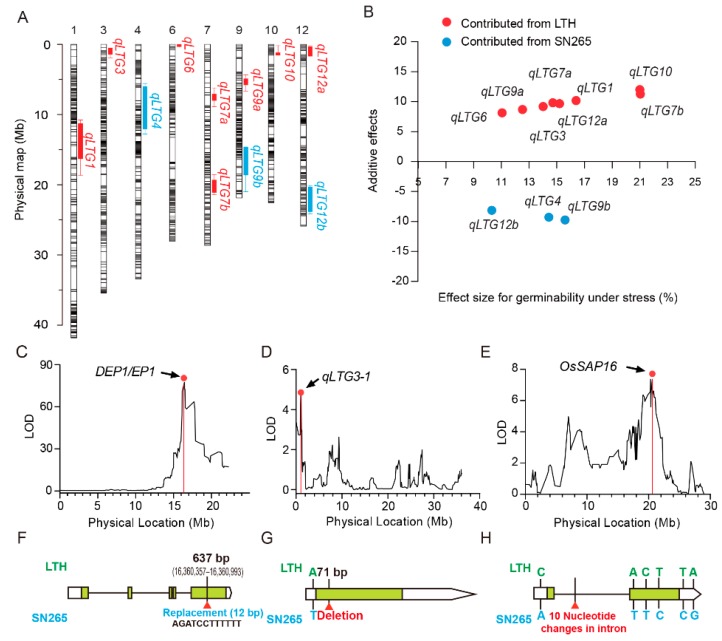
(**A**). QTL scan using whole-genome sequencing and a sequence comparing the panicle curvature and low-temperature germinability QTLs near the previously identified genes. Curves indicate the chromosome locations (Mb) and the low-temperature germinability (LOD) values of the detected QTLs. Arrowheads represent the relative genetic positions of the candidate genes. (**A**). Genomic locations of the 11 QTLs with strong effects for low-temperature germinability (LOD > 3) identified in the RIL population; (**B**). Plots of the additive effect and the allele effect of the 11 QTLs. (**C**). A plot of the LOD values of the panicle curvature. (**D**). A plot of the LOD values of the low-temperature germinability on chromosome 3. (**E**). A plot of the LOD values of low-temperature germinability on chromosome 7. (**F**). Sequence comparisons of the *DEP1* region between LTH and SN265. (**G**). Sequence comparisons of the *qLTG3-1* region between LTH and SN265. (**H**). Sequence comparisons of the *OsSAP16* region between LTH and SN265.

QTL mapping for the typical panicle trait named *Dense and Erect Panicle1* (*DEP1*) was performed to estimate the accuracy and mapping resolution ability of the respective bin maps and the RILs. Careful observations were taken for the panicle curvature of the 144 RILs lines 25 days after the start of heading, whereas the erect-type panicle lines remained erect. One QTL was identified on chromosome 9, with a peak interval of 16.4 Mb, as shown in Figure 3C. The QTL peak was located on the previously characterized and cloned *DEP1/EP1* gene. Sequence comparisons of the *DEP1* region between LTH and SN265 illustrated the replacement of a 637-bp in the middle of exon-5 by a 12-bp sequence (Figure 3F), which caused a frame shift mutation, as described previously [28].

### 2.4. QTL Analysis of Low-Temperature Germinability

After combining the bin-map and phenotyping data of each RIL line, scanning of the LTG QTL was carried out. A total of 11 QTLs were identified on chromosome 1 (11.48–19.34 Mb), chromosome 3 (0.00–1.31 Mb), chromosome 4 (6.10–11.41 Mb), chromosome 6 (0.34–0.74 Mb), chromosome 7 (6.98–9.21 Mb; 18.93–21.76 Mb), chromosome 9 (5.91–6.83 Mb; 14.91–21.38 Mb), chromosome 10 (1.20–1.60 Mb), and chromosome 12 (0.86–2.35 Mb; 21.05–25.16 Mb). The confidence intervals associated with these QTLs spanned the genomic physical position from 0.07 to 7.86 Mb compared with the reference genome of rice (Table 2, Figure 3A). Five QTLs showed relatively small confidence intervals of less than 1.5 Mb, including *qLTG-3* (1.31 Mb), *qLTG6* (0.40 Mb), *qLTG-9a* (0.92 Mb), *qLTG-10* (0.40 Mb), and *qLTG-12a* (1.49 Mb). Among these QTLs, phenotypic variation ranged from 10.30% to 21.04% (Table 2, Figure 3B). Overall, five QTLs depicted the explained phenotypic variation exceeding 15%, and two major QTLs were detected: *qLTG7b*, found on chromosome 7, with 21.04% phenotypic variation and 7.39 of the LOD value; and *qLTG10*, located on chromosome 10 and comprised of 21.00% phenotypic variation with a 7.38 LOD value. Furthermore, eight QTL alleles, *qLTG1*, *qLTG3*, *qLTG6*, *qLTG7a*, *qLTG7b*, *qLTG9a*, *qLTG10*, and *qLTG12a*, had a positive effect inherited from LTH. The other three QTL alleles, *qLTG4*, *qLTG9b*, and *qLTG12b*, had a positive effect inherited from SN265 (Figure 3B). Based on the high-resolution bin map, two QTLs, *qLTG3* and *qLTG7b*, were localized to the chromosome intervals that subsumed the cloned LTG genes. The QTL named *qLTG3* was located in the interval of the LTG gene described as *qLTG3-1*, with a 4.71 LOD value and 14.02% phenotype variance [9]. The QTL named *qLTG7b* had a 7.39 LOD value and 21.04% phenotypic variation and was located on *OsSAP16*, a rice LTG gene that has already been identified by the association study method [10]. PCR amplification and sequencing were conducted to screen out the causal polymorphisms of *qLTG3-1* and *OsSAP-16*, which determined that LTH has two cold-tolerant genes (Figure 3E,F). Additionally, these results also indicate that the data used for scanning QTLs are entirely effective (Figure 3D,E,G,H).

### 2.5. Fine Mapping and Candidate Gene Prediction for qLTG6

**Figure 4:**
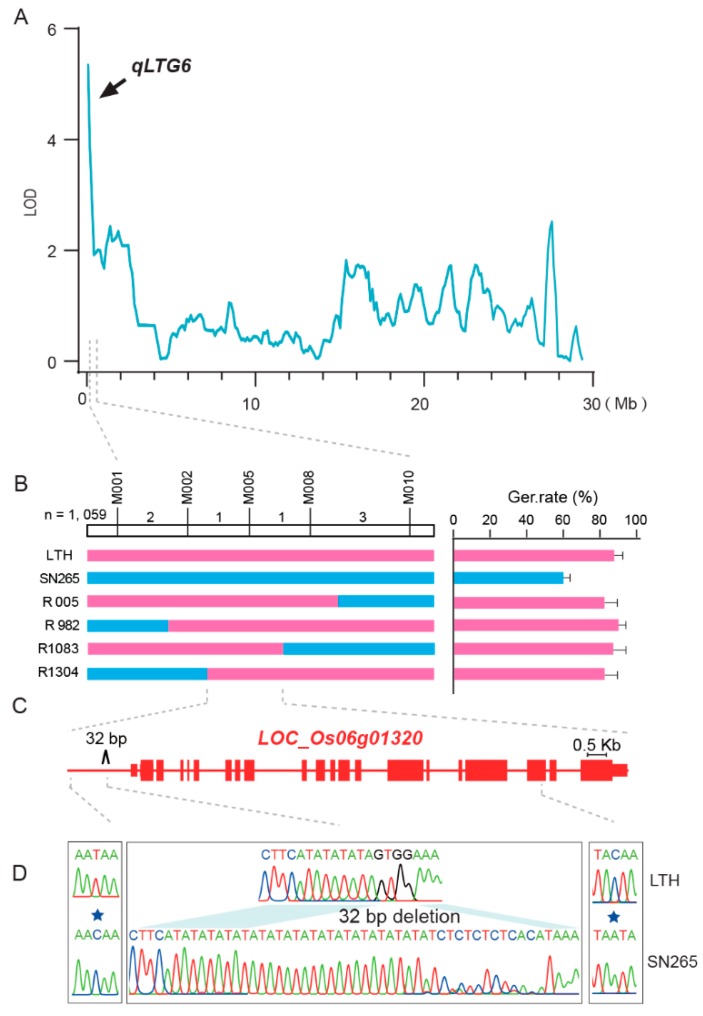
Identification of the candidate gene of the QTL, *qLTG6*. (**A**). Linkage analyses of the QTL, *qLTG6*; (**B**). Graphical representation of recombinants in the RILs of germinability under low temperatures, refining the location of *qLTG6* in an interval defined by bin markers; (**C**). The identification of the candidate gene, *LOC_Os06g01320*, which encodes a chromodomain, helicase/ATPase, and DNA-binding domain (CHD)-related (CHR) proteins, CHR723; (**D**). Validation of the mutation of *LOC_Os06g01320* by PCR sequencing.

To identify the possible novel genes for low germination within the QTLs, *qLTG6* was thoroughly analyzed because it has a high LOD value and relatively small genetic intervals (Figure 4A, Table 2). *qLTG6* was first identified within a 400 kb interval. Then, further high-resolution mapping of *qLTG6* was carried out using four key homozygous recombination lines from 1,569 segregating population plants and newly developed markers, as shown in Table 3. Finally, *qLTG6* was localized to a region of a 45.8 kb physical interval between markers M002 and M008 via the progeny testing of key homozygous recombinant lines (Figure 4B). Overall, there was the prediction of about seven genes in the target region (Table 4). A re-sequencing data analysis of the delimited region only detected differences in the gene *LOC_Os06g01320* (Figure 4C). To confirm and estimate these differences, PCR-based sequencing was conducted to analyze the gene body and the 3 kb promoter of *LOC_Os06g01320*, which revealed a 32 bp deletion and a T–C transition in the promoter. In the gene body, a C–A transition was also detected in the 18th exon, causing a substitution of Thr to Asn in LTH (Figure 4D).

### 2.6. Expression Analysis of LOC_Os06g01320

**Figure 5:**
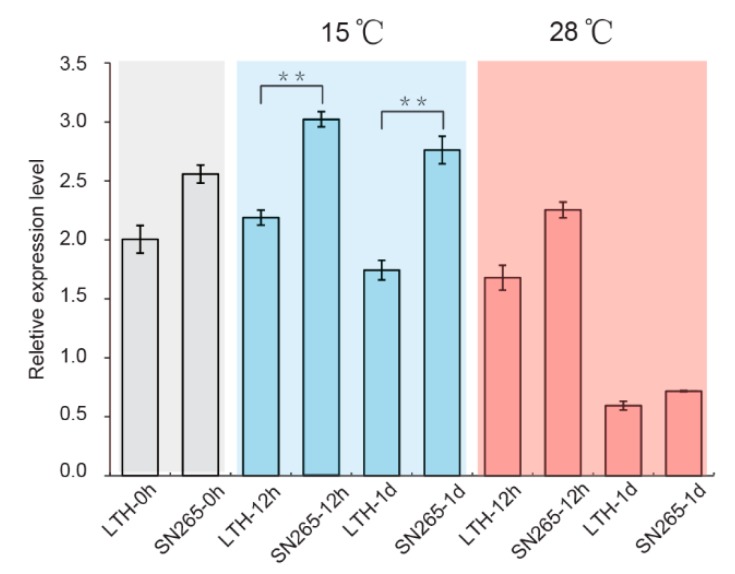
The expression analysis of the candidate gene of *qLTG6*. Relative expression of LOC_Os06g01320 at the germination stage under 15 °C and 28 °C in the LTH and SN265, respectively. ** *p* < 0.01, using Student’s *t*-test. Bar SEM, *n* = 3.

Both an SNP and deletion were detected at the promoter of *LOC_Os06g01320* in LTH, which suggests that expression might vary between parents. For further investigations on these variants, qPCR analyses were executed to evaluate the expression patterns of *LOC_Os06g01320* in two parents. During seed germination under optimal conditions at 28 °C, the expression levels of *LOC_Os06g01320* decreased in the LTH and SN265 from 0 h to 1 d (Figure 5), which indicates that *LOC_Os06g01320* is necessarily required to be repressed during seed germination. Under the conditions of low-temperature stress with a temperature range around 15 °C, the expression levels of *LOC_Os06g01320* in LTH were significantly lower than SN265, both at 12 h and at 1 d. After conducting a further refinement, these results showed that LTH has lower expression levels of *LOC_Os06g01320* than SN265, which could reduce the negative regulation of gene expression and promote seed germination under low-temperature stress.

## 3. Discussion

In recent years, direct-seeded rice has received much attention in Asia because of its time and labor savings and low input demand as an alternative to conventional rice systems. However, the long-term cultivation method of seedling-transplantation has led to a loss of expressions of some low-temperature-tolerant genes, which are usually expressed at the germination stage. Poor germinability remains one of the major problems [5,21,29,30]. Therefore, screening cultivars with high germination abilities under low-temperature stress is necessary to sustain rice yield and ensure the application of direct seeding cultivation in the NEC region. In the current study, the *japonica* landrace variety, LTH, a locally well-adopted cultivar originating from the far Southwest province of China, was selected since it shows a high germination rate under low-temperature conditions. The germination of LTH began two days after the start of incubation at a temperature of 15 °C and took five more days to reach a germination rate of almost 90%. Screening of the low-temperature germination ability of 135 cultivars from the NEC was then done, which revealed that no cultivar was more tolerant to low-temperature stress than LTH. Therefore, the identification of tolerant genes during germination under low-temperature stress in LTH has important scientific value for improving the low-temperature germination ability of rice in the NEC.

In this study, high-throughput genotyping was employed through whole-genome resequencing, and bin map construction was carried out for QTL mapping. A total of 11 QTLs were identified on Chr.1, Chr.3, Chr.4, Chr.6, Chr.7, Chr.9, Chr.10, and Chr.12. By comparing the positions of the QTLs, 19 previously identified LTG QTLs were found to be near 10 QTLs, except for *qLTG9a*, as presented in Table 5. The *qLTG-1* was mapped near the *qCTGERM1-5* region for LTG [31]. The QTL location of *qLTG-3* was very close to that of the cloned QTL *qLTG3-1* [9]. The QTLs of *qLTG4*, *qLTG6*, and *qLTG10* were as also found by Teng et al. [32], Ji et al. [33] and Shakiba et al. [31] to enhance low-temperature germinability. *qLTG7a* was mapped near *qCTGERM7-1* [31], *qLTG-7* [34], *qGR-7*, and *qGI-7* [35]. Furthermore, *qLTG7b* was found to be mapped near *qCTGERM7-4* [31], *qLTG-7* [36], and *OsSAP16* [10]. At the germination stage, there was a correlation between the cold tolerance of *qLTG9b* and the *qLTG-9* region [32,33]. *qLTG12a* was mapped near *qLTG-12a* [37] and *qLTG-12* [38]. *qLTG12b* was identified near the region of *qCTGERM12-1* [31], *qGR-12* [10], *qLTG-12* [36], and *qLTG-12* [39]. The above comparison results reflect the accuracy of this study, as well as the complexity of cold tolerance during germination. Moreover, this study also suggests that LTH’s strong low-temperature germination ability was acquired by accumulating more cold-tolerant genes. These results not only strengthen the findings of previous studies but also reflect the complexity of low-temperature germination in accelerating the breeding programs for enhanced cold-tolerance among rice cultivars.

Among the 11 QTLs identified in this study, *qLTG-6* was ultimately narrowed down to the 45.80 kb region (Figure 4B). Currently, seven genes have been observed in the target region (Table 5) where *LOC_Os06g01250* is found to encode the protein named cytochrome P450. *LOC_Os06g01260* has functional activities in encoding Glutathione gamma-glutamyl cysteinyl transferase-1. Moreover, *LOC_Os06g01280* encodes a retrotransposon protein. *LOC_Os06g01270* and *LOC_Os06g01290* encode a protein that has yet to be discovered. *LOC_Os06g01304* encodes Spotted Leaf 11, whereas *LOC_Os06g01320* encodes chromodomain, helicase/ATPase, and DNA-binding domain proteins. Considering the organ specificity in gene expression and molecular function information, it is difficult to ensure that a gene is a target gene. According to the sequencing data, this study identified only the C–A transition in the 18th exon of *LOC_Os06g01320*, which is predicted to result in the substitution of Thr to Asn in LTH. We also found a 32 bp deletion and a T–C transition in the promoter region. In addition, the expression level of *LOC_Os06g01320* in LTH was found to be lower than that of SN265 under lower temperatures. This lower expression level could be associated with seed germination at low-temperature stress. The sequence and gene expression data suggest that *LOC_Os06g01320* might be the most plausible prospect for *qLTG-6*, but the current evidence remains insufficient and will require us to carry out genetic modification complementation or gene editing verification.

## 4. Materials and Methods

### 4.1. Plant Materials

The 144 *japonica* rice RIL population was built via the single-seed descent method from a cross between Shennong265 (SN265) and Lijiangxintuanheigu (LTH). The SN265 is a locally well-adopted and super high yielding cultivar in Liaoning province, NEC, whereas LTH is a landrace from the Yunnan Province, in the far Southwest of China. These RILs were planted at the agricultural farm of institute of Crop Cultivation and Tillage, Heilongjiang Academy of Agricultural Sciences. The DNA of F11 RIL generations was isolated for genotyping with the specified protocols. One residual heterozygous plant was selected in the F6 population for the fine mapping of *qLTG6*. It had already been observed through the genotyping of 114 DNA markers that the *qLTG6* region of this selected plant is heterozygous, and other chromosome regions are homozygous [40]. A total of 1,569 segregating individuals were developed from the selected heterozygous plant.

### 4.2. Preparation of Seeds for the Germination Test

The RILs and their parents were grown in the experimental fields in 2017, where the nursery sowing date was 15th April, and transplanting was done on 16th May, with one seedling per hill. Sixty plants of each line were planted in 4 rows, with a plant to plant distance of 13.3 × 30 cm. The planting of the segregated *qLTG6* population was performed during the rice-growing seasons of 2018. The sowing and transplanting dates were 18th April and 21st May, respectively. The transplanting standards were kept consistent with those of the RILs. Field management practices were done according to the most followed agricultural practices of local farmers. The nitrogen (N), phosphorus (P), and potassium (K) fertilizers, in the form of Urea, single superphosphate, and Murate of Potash, were applied at rates of 120, 60, and 60 kg/ha, respectively. Rice harvesting was done on 30 September, and the plants were retained to dry them for three months. Immature and unfilled grains were removed to obtain high quality grain, which was stored at 5 °C to maintain the relative humidity around 10%.

### 4.3. Evaluation of Germinability under Cold Stress

The evaluation of the germination ratio under cold stress was performed following standard protocol. The seeds broke out of dormancy at 50 °C after 48 h. Further matured and fully-filled grains were sterilized with 0.1% mercury chloride solution for about 10 min, rinsed with tap water 3 to 4 times, and deionization was performed with deionized water 3 to 4 times. One hundred grains per line were placed on a filter paper in a petri dish, and 10 mL of distilled water was added. The petri dishes were then put in an incubator at the recommended temperature of 15 °C. To avoid any kind of reactions and contaminations, the tap water was changed every two days. The germination of the seeds was noted carefully every day. The germination rate on the 6th day was used for QTL mapping because of the large differences observed between the two parents. The germination was noted three times for each line.

### 4.4. DNA Extraction, Re-Sequencing, and SNP Calling

The extraction of the genomic DNA of the two parents and each RIL was performed using a modified CTAB method [41]. Biomarker Technologies were used for the re-sequencing of the parents and RILs. The procedure was performed according to Jiang et al. [41]. The short read alignment was done as described by Li and Durbin [42]. Straining of the low-quality data was performed to produce better-quality mapping. The clean data were then aligned to the Nipponbare reference genome (Os-Nipponbare-Reference-IRGSP-1.0) [43] using the BWA software [42]. The calculations of the sequencing coverage and depth were performed through Samtools [44]. Then, the Genome Analysis Toolkit (GATK) was used to detect the SNPs with default parameters [45]. The accession number raw sequence data obtained in our study have been deposited in the NCBI Short Read Archive, with accession number PRJNA587802.

### 4.5. Genotyping and Construction Bin Map

The estimation of genotype calling parameters, determination of the recombination breakpoint, and construction of the bin map were carried out with minor modifications via the method of Sliding Window, as described by Huang et al. [19]. The genotype of each window was determined by the SNP ratio between the two parents; if the SNP ratio of SN265 to LTH was 15:5 or higher, the genotype was considered to be a homozygous SN265 genotype, if the ratio was lower, then it was designated as a homozygous LTH genotype. Moreover, if the SNP ratio between the two parents was between 5:15 and 15:5, the genotype was recognized as heterozygous. The genotype calling parameters were performed according to the methods described by Song et al. [22]. Adjacent 15 kb segments with the same genotypes were merged as one bin marker [46]. The linkage map was built using the *est.map* function with the R/qtl software [47].

### 4.6. QTL Mapping for Low-Temperature Germinability

In this study, we used the composite interval method (CIM) in the R/qtl package [47] to detect the QTLs for LTG. The threshold level of CIM was ensured by 1000 permutations, and the QTL confidence interval was estimated by using a 1.5 LOD-drop from the peak LOD. The germination rate (%) after 6 days under controlled temperature conditions of 15 °C was used for the QTL analysis.

### 4.7. qRT-PCR and Expression Analysis

The extraction of the total RNA was carried out with a TRIzol reagent. The first-strand of cDNA was reverse transcribed by using a TransScript II First-Strand cDNA Synthesis SuperMix kit (Transgen). The qRT–PCR analysis was executed by using the kit named SYBR FAST qPCR (KAPA). The qPCR primer pairs sequences for *LOC_Os06g01320* were 5′-CAAAAAAAAAGACAATAAGGTGGA-3′ (forward) and 5′-CAGACATTGCTTACCCTTATTTATTTT-3′ (reverse). EF-1 alpha was used as an internal control, and the sequencing was 5′-GCACGCTCTTCTTGCTTTCACTCT-3′ (forward) and 5′-AAAGGTCACCACCATACCAGGCTT-3′ (reverse).

## Figures and Tables

**Table 1:** Characteristics of the high-density genetic map derived from a cross between LTH and SN265.

Chr. ^a^	No. Markers ^b^	Genetic Distance (cm)	Physical Distance (Mb)	Avg Distance between Markers (cm/kb)	<1 Mb Gap	Min. Gap (kb)	Max. Gap (Mb)
1	254	338.65	42.93	1.33/169.03	246	15.27	3.16
2	254	227.11	31.61	0.89/124.46	252	15.47	3.45
3	313	284.80	36.14	0.91/115.45	310	15.06	1.57
4	281	300.18	34.29	1.07/122.03	275	15.63	1.91
5	239	269.15	29.68	1.13/124.19	235	15.32	1.08
6	247	181.10	28.63	0.73/115.92	244	15.53	3.63
7	187	217.94	29.23	1.17/156.34	184	17.18	1.46
8	238	193.86	28.12	0.82/118.15	237	15.02	0.99
9	188	111.32	22.30	0.59/118.61	185	15.89	1.92
10	234	199.84	22.99	0.85/98.23	230	15.77	1.27
11	186	323.32	28.49	1.74/153.18	183	15.01	3.48
12	207	192.85	26.70	0.93/130.43	202	15.63	2.33

^a^ Chr., indicates chromosome; ^b^ No. markers, the number of markers on the chromosome.

**Table 2:** Characteristics of the high-density genetic map derived from a cross between LTH and SN265.

QTL	Chr. ^a^	Peak		QTL Interval			LOD ^c^	Var (%) ^d^	Add. ^e^	Positive Allele
		Pos. (cm)	Pos. (Mb) ^b^	Linkage (cm)	Physical (Mb)	Location Interval (cm/Mb)				
*qLTG1*	1	116.34	16.90	103.19–128.81	11.48–19.34	25.62/7.86	5.59	16.40	10.16	LTH
*qLTG3*	3	3.95	1.10	0.00–10.78	0.00–1.31	10.78/1.31	4.71	14.02	9.18	LTH
*qLTG4*	4	54.58	6.27	36.64–77.67	6.10–11.41	41.03/5.31	4.85	14.43	−9.30	SN265
*qLTG6*	6	0.18	1.34	0.18–1.07	0.34–0.74	0.89/0.40	3.64	11.05	8.13	LTH
*qLTG7a*	7	104.99	8.88	87.28–107.84	6.98–9.21	20.56/2.23	4.98	14.72	9.80	LTH
*qLTG7b*	7	152.12	20.32	148.13–162.80	18.93–21.76	14.67/2.84	7.39	21.04	11.26	LTH
*qLTG9a*	9	4.31	6.07	4.35–8.38	5.91–6.83	4.03/0.92	4.17	12.53	8.68	LTH
*qLTG9b*	9	79.24	15.27	71.69–102.85	14.91–21.38	31.16/6.47	5.29	15.60	−9.76	SN265
*qLTG10*	10	13.93	1.60	8.55–14.06	1.20–1.60	5.51/0.40	7.38	21.00	12.00	LTH
*qLTG12a*	12	6.17	0.87	4.72–16.25	0.86–2.35	11.53/1.49	5.14	15.20	9.65	LTH
*qLTG12b*	12	131.25	22.79	111.25–145.46	21.05–25.16	34.21/4.11	3.41	10.30	−8.12	SN265

^a^ Chr., chromosome; ^b^ Positions in the linkage map (unit: Mb); ^c^ Logarithm (base 10) of the odds for the corresponding QTL peak; ^d^ Percentage of the phenotypic variation explained by the corresponding QTL; ^e^ Additive effect of the corresponding QTL.

**Table 3:** The markers developed for the fine mapping of *qLTG6* on chromosome 6.

Molecular Marker	Primer Sequence (5′→′)
M001	CTTCGCACTCCAGTCGCTCTCC GTTGAGGAGGTGTATGGGCTTGG
M002	AGCTCACCAGGGACAACATCAAGG TTAACCAGCTCCGCCAGCATCC
M005	CGCCACTGATCGATCTCCTCTCC CGAGCTGGCCTTCTTCCTTGG
M008	AATTGATGCAGGTTCAGCAAGC GGAAATGTGGTTGAGAGTTGAGAGC
M010	TGTTGGATTGGAATCGGAAAGC CTCTGCTGTGCTGTGCTGCTAGG

**Table 4:** Candidate genes in the 45.8 kb target region corresponding to *qLTG6.*

Name	Location	Protein
*LOC_Os06g01250*	163205–165539	Cytochrome P450
*LOC_Os06g01260*	167364–174331	Glutathione gamma-glutamylcysteinyltransferase 1
*LOC_Os06g01270*	178580–178343	Expressed protein
*LOC_Os06g01280*	180215–181423	Retrotransposon protein
*LOC_Os06g01290*	182104–184623	Expressed protein
*LOC_Os06g01304*	185692–191452	Spotted leaf 11
*LOC_Os06g01320*	195018–208583	Chromodomain, helicase/ATPase, and DNA-binding domain (CHD) proteins

**Table 5:** Comparison of QTL positions on the rice genome.

QTL	Chr. ^a^	QTL interval	Prior near QTLs Location	Reference
		Physical (Mb)	Physical (Mb)	
*qLTG1*	1	11.48–19.34	*qCTGERM1-5* (12.71)	[31]
*qLTG3*	3	0.00–1.31	*qLTG3-1* (0.22)	[9]
*qLTG4*	4	6.10–11.41	*qLTG-4* (6.58–13.64)	[32]
*qLTG6*	6	0.34–0.74	*qLTG-6* (0.65–2.69)	[33]
*qLTG7a*	7	6.98–9.21	*qCTGERM7-1* (10.46–10.65)	[31]
*qLTG7b*	7	18.93–21.76	*qLTG7* (20.16–22.55)	[34]
			*qGR-7* and *qGI-7*(20.35–21.59)	[35]
			*qCTGERM7-4* (19.59–20.26)	[31]
			*qLTG-7* (16.88–22.52)	[36]
			*OsSAP16* (22.93)	[10]
*qLTG9a*	9	5.91–6.83		
*qLTG9b*	9	14.91–21.38	*qLTG-9* (12.29–18.90)	[32]
			*qLTG-9* (11.81–15.32)	[33]
*qLTG10*	10	1.20–1.60	*qCTGERM10-1* (1.40–1.53)	[31]
*qLTG12a*	12	0.86–2.35	*qLTG12a* (0.75)	[37]
			*qLTG-12* (2.43–3.19)	[38]
*qLTG12b*	12	21.05–25.16	*qCTGERM12-1* (24.89–24.90)	[31]
			*qGR-12*(22.78–25.15)	[10]
			*qLTG12* (24.52–25.08)	[36]
			*qLTG-12* (24.52–25.08)	[39]

^a^ Chr., chromosome.
